# Plant-Insect Interactions

**DOI:** 10.3390/plants11091140

**Published:** 2022-04-22

**Authors:** Francisco Rubén Badenes-Pérez

**Affiliations:** Instituto de Ciencias Agrarias, Consejo Superior de Investigaciones Científicas, 28006 Madrid, Spain; fr.badenes@csic.es

## 1. Introduction

The central part of the study of plant-insect interactions comes from our quest for knowledge on why and how these interactions occur. Within the broad topic of plant-insect interactions, insect pest management and insect plant pollination are among the most relevant research areas because of the economic impact that they can have on crop yield [1,2,3,4,5]. This Special Issue presents a collection of papers dealing with basic and applied topics in plant-insect interactions, but showing the relative importance that pest management and pollination have in this field.

## 2. Key Messages

Host-plant resistance is a prominent part of integrated pest management [6]. In Brassicae, glucosinolates play a key role in host-plant resistance [7]. Three papers in this Special Issue deal with glucosinolates and host-plant resistance to insect pests [8,9,10]. Genotypes of kale *Brassica oleracea* L. var. *acephala* (Brassicaceae) that have different glucosinolate content can differ in glucosinolate induction and resistance to the cabbage moth, *Mamestra brassicae* L. (Lepidoptera: Noctuidae) [8]. Another generalist lepidopteran, the cotton bollworm *Helicoverpa armigera* Hübner (Lepidoptera: Noctuidae), can metabolize glucosinolates via conjugation to glutathione, however a high content of nitrogen and sulphur amino acid contents in the plant facilitates this process [9,11]. *Helicoverpa armigera*, however, does not seem to improve its performance on plants containing glucosinolates after selection for glucosinolate adaptation [10]. Despite this, *H. armigera* has a pest status in Brassicaceae, and this is also due to other factors, such as the damage that this insect causes to plant reproductive structures [12], insecticide resistance [13], and suppression of natural enemies by insecticide use [14].

In gladiolus *Gladiolus hybridus* L. (Iridaceae), host-plant resistance to western flower thrips *Frankliniella occidentalis* Pergande (Thysanoptera: Thripidae) can be conferred by morphological factors, such as the density of epicuticular papillae, and by chemical factors, such as content of triterpenoid saponins [15]. Triterpenoid saponins have also been suggested as being involved in resistance to *F. occidentalis* in a different plant, *Barbarea vulgaris* R. Br. (Brassicaceae) [16].

Insect population dynamics are greatly affected by weather conditions [17,18,19]. Bažok et al. show that corn damage caused by the first generation of European corn borer *Ostrinica nubilalis* Hübner (Lepidoptera: Crambidae) is positively correlated with air temperature and negatively correlated with air humidity [20]. Fertilizer use can also affect insect preference and development on plants [21,22,23]. In this regard, Li et al. show that the white-backed planthopper *Sogatella furcifera* (Horváth) (Hemiptera: Delphacidae) prefers plants grown in substrate containing high content of nitrogen fertilizer, developing also faster and having a longer lifespan when feeding on plants grown under high nitrogen conditions [24]. Endophytic microorganisms can also influence plant-insect interactions and the plant response to herbivory [25,26]. *Bacillus subtilis* 26D Cohn secretes cytokinins that help plantlets of potato *Solanum tuberosum* L. (Solanaceae) recover after herbivory by the Colorado potato beetle *Leptinotarsa decemlineata* Say (Coleoptera: Chrysomelidae) [27].

Pollinators and natural enemies of pests are essential for food production [28,29,30,31,32]. Because of this, insectary plants can be used for conservation biological control and pollinator conservation [33,34]. The paper by Kati et al. shows that some wildflowers planted in the field margins of tomato fields attract high numbers of wild bees, honeybees, and parasitoids [35]. Their research also shows that flower cover is correlated with the abundance of wild bees and honeybees. Tomato is one of the crops that requires insect pollination in order to maximize yield [36] and, therefore, the presence of wildflowers that attract pollinators is likely to have a positive effect on tomato yield. The paper by Kati et al. also includes some plant species that have not been previously tested as insectary plants. The study of insectary plants often focuses on few plant species that have often been proven to fulfill their purpose, such as buckwheat *Fagopyrum esculentum* Moench (Polygonaceae), *Lobularia maritima* L. Desv. (Brassicales), and *Phacelia tanacetifolia* Benth. (Boraginaceae) [37,38,39]. In terms of understanding the mechanisms of attraction of flowers to pollinators, Giuliani et al. show that glandular trichomes and monoterpene VOCs could be involved in attracting pollinators to two *Salvia* spp., *S. blepharophylla* Brandegee ex Epling and *S. greggii* A. Gray (Lamiaceae) [40].

## 3. Future Directions

Plant-insect interactions include a tremendous diversity of relationships deserving further research [41,42]. In these interactions, plant secondary metabolites can play an important role [43,44,45]. In this Special Issue, different chemical compounds were studied for their role in host-plant resistance. However, identification of additional chemical compounds involved in plant-insect interactions continues to be of importance. For example, in Brassicaceae, a lot of the research conducted in plant-insect interactions in the context of chemical ecology has been conducted with glucosinolates [7,46,47]. Future research is necessary to study the role that other plant secondary metabolites may play in plant-insect interactions that have so far been studied mostly by measuring glucosinolate content. The paper by Jeschke et al. [9] indicates that content of other plant compounds, such as amino acids, can affect the ability of the insect to detoxify plant secondary metabolites. Furthermore, the identification of the two unidentified triterpenoid saponins that seem to confer gladiolus resistance to *F. occidentalis* [15] could uncover additional compounds of importance in host-plant resistance to this and other herbivores. It should also be investigated if there are ontogenetic and seasonal changes in saponin content in gladiolus. In other plant species, based on the changes observed in insect resistance, ontogenetic, phenological, and seasonal changes in saponin content seem to occur [48,49,50]. Moreover, the effect of bacteria on plant repair mechanisms after insect herbivory and plant-insect interactions in general also deserves being subject to further research. In terms of pollination, after having tested the effect of wildflowers on attraction to pollinators and natural enemies, as in the case of the margins of tomato fields [35], further research should measure how the presence of wildflowers affects fruit quality and yield.
